# Transferable resistance in multiresistant Gram-negative bacteria isolated from hemocultures in Slovakia

**DOI:** 10.1186/2047-2994-4-S1-P127

**Published:** 2015-06-16

**Authors:** N Kulkova, L Michalikova, JS Brnova, V Krcmery

**Affiliations:** 1 Laboratory of Molecular Microbiology, St. Elisabeth University, Bratislava, Slovakia; 2 Department of Laboratory Medicine, School of Health Sciences and Social Work, Trnava University, Trnava, Slovakia

## Introduction

Antimicrobial resistance can be transferred between bacteria, and their plasmid-encoded resistant genes can be next transferred to other pathogens.

## Objectives

The aim of this study was to assess the character of transferable resistance in multiresistant clinical isolates of Gram-negative bacteria from blood cultures in Slovakia.

## Methods

This multicentre study was performed in November 2011 – January 2013. Altogether, 269 isolates of GNB from positive blood cultures of septic patients from hospitals in Slovakia were analysed. Transferability of resistance–determinants was assessed by phenotypic methods with conjugational experiments. Strains of rifampin-resistant *Escherichia coli* 3110, rifampin-resistant *Proteus mirabilis* P38, rifampin-resistant *Pseudomonas aeruginosa* 1008, and rifampin resistant *Pseudomonas aeruginosa* 1670 were used as recipient strains. *Escherichia coli* strain ATCC 25922 was used as a control strain.

## Results

Laboratory analysis of transferable resistance was performed with 213 clinical isolates, excluding 14 isolates for selectable resistance of rifampicin and nalidixic acid. Transferable resistance was confirmed in 61 isolates (28,6%), of which 18 (29,5%) in *Klebsiella pneumoniae*, 5 (8,2%) in *Proteus mirabilis* and in 7 (11,5%) other species. Cefotaxime (36; 59%), ceftazidime (28; 45,9%) and aztreonam (23; 37,7%) resistances were the most frequently transferred resistotypes. Transfer only the one determinant of resistance was observed in 26 isolates (42,6%) and the multiple transfer in 35 isolates (57,4%). The most frequent recipient of antibiotic resistance determinants was strain Escherichia coli 3110 with 52 transfers (85,2%), followed Proteus mirabilis P38 (30 transfers; 49,2%) and Pseudomonas aeruginosa (9 transfers; 14,8%). Interspecies transfer among the resistant bacteria was observed in 18 isolates (29,5%).

## Conclusion

In this study, we described high proportion of transferable resistance among multiresistant clinical isolated of Gram-negative bacteria in Slovakia.

## Disclosure of interest

None declared.

